# The uniqueness of the human brain: a review

**DOI:** 10.1590/1980-5764-DN-2023-0078

**Published:** 2024-04-15

**Authors:** José Eymard Homem Pittella

**Affiliations:** 1 Universidade Federal de Minas Gerais, Faculdade de Medicina, Departamento de Anatomia Patológica e Medicina Legal, Belo Horizonte MG, Brazil.

**Keywords:** Brain, Anatomy, Neurosciences, Human Evolution, Pan troglodytes, Encéfalo, Anatomia, Neurociências, Evolução Humana, Pan troglodytes

## Abstract

The purpose of this review is to highlight the most important aspects of the anatomical and functional uniqueness of the human brain. For this, a comparison is made between our brains and those of our closest ancestors (chimpanzees and bonobos) and human ancestors. During human evolution, several changes occurred in the brain, such as an absolute increase in brain size and number of cortical neurons, in addition to a greater degree of functional lateralization and anatomical asymmetry. Also, the cortical cytoarchitecture became more diversified and there was an increase in the number of intracortical networks and networks extending from the cerebral cortex to subcortical structures, with more neural networks being invested in multisensory and sensory-motor-affective-cognitive integration. These changes permitted more complex, flexible and versatile cognitive abilities and social behavior, such as shared intentionality and symbolic articulated language, which, in turn, made possible the formation of larger social groups and cumulative cultural evolution that are characteristic of our species.

## RESUMO

Esta revisão se propõe a relatar os aspectos mais importantes da singularidade anatômica e funcional do cérebro humano. Para isso, faz-se uma comparação entre o nosso cérebro e os de nossos parentes evolutivos mais próximos (chimpanzés e bonobos) e os ancestrais humanos. Durante a evolução humana ocorreu aumento absoluto do tamanho do cérebro e do número de neurônios corticais cerebrais, maior grau de lateralização funcional e assimetria anatômica cerebral, citoarquitetura cortical mais diversificada e aumento das redes neurais intracorticais e do córtex cerebral para as estruturas subcorticais acompanhada de mudança em direção ao investimento de redes neurais na integração multissensorial e sensório-motora-afetiva-cognitiva. Essas mudanças possibilitaram capacidades cognitivas e comportamentos sociais complexos, flexíveis e versáteis, destacando-se a intencionalidade compartilhada e a linguagem articulada simbólica, que permitiram a formação de grupos sociais maiores e a evolução cultural cumulativa característica de nossa espécie.

## INTRODUCTION

The purpose of this review was to inform and comment on some anatomical and functional aspects of the human brain — nature's most refined structure and, therefore, the central component of human identity. Understanding the structure and function of the human brain is essential for answering the question of why we are the species we are^1^.

Over the millions of years of evolution of our brain, structures and neural networks that were present in the brains of our human ancestors, as well as in the brains of our closest evolutionary relatives — great apes, in particular chimpanzees and bonobos — have been incorporated and changed. Therefore, information on the brains of these preceding species is necessary to help provide answers to the question of human identity from a neuroscience perspective.

What exactly makes our human brain different from the brains of our human ancestors and those of chimpanzees and bonobos? The questions to be asked are "is there is any uniqueness in the human brain? If so, what is it and at what level — macroscopic, microscopic, or both — is it present? Is there any special anatomical arrangement or neural network in the connectome? Is there a greater number of brain neurons or a larger expansion or distinct architectural organization of the cerebral cortex in humans? On the other hand, if there is no uniqueness, are the differences between the human brain and the brains of our ancestors and great apes only quantitative?".

These are the questions to be answered in this review, based on the advances in neuroscience over the past decades and years. Throughout this review, the term "brain" is used instead of encephalon, as the brain represents the largest part of the encephalon (which includes, in addition to the brain, the brain stem and the cerebellum). Thus, the topics described herein refer to brain structures and functions. For a better understanding of the text, the following topics are addressed herein^2^:

- Brain size and weight;
- Number of cerebral cortex neurons;
- Energy consumption by the brain;
- Cerebral functional and anatomical asymmetry;
- Cerebral cortex expansion;
- Prefrontal cortex;
- Connectome.

### Brain size and weight

In terms of size, the human brain is three to four times larger than the brains of great apes. The frontal lobe is the largest, followed by the parietal, temporal, and occipital lobes^3-5^. Another difference between humans and great apes refers to frontal lobe position. Due to its large expansion in humans, the frontal lobe became positioned above the orbits (supraorbital position), leading to humans' high, elevated forehead. In great apes, the frontal lobe is situated behind the orbits (retro-orbital position), hence their low, slanted forehead. In addition, because of its large expansion, the most anterior portion of the frontal lobe, referred to as the prefrontal cortex, has acquired a more rounded contour in humans, whereas it is more pointed in large apes, a little less so in bonobos. Several factors contribute to determining brain size in different species, such as the number of neurons and glial cells, neuron body size, dendritic arborization, axonal diameter, blood vessels, and extracellular space.

In general, brain size varies according to the animal's size and weight. The bigger the animal, the larger the size and weight of its brain tend to be, as it needs to control its body and make it function. An increase in the relative brain size of a species, with brain volume exceeding that expected for the species, will allow greater cognitive capacity, as observed in humans.

On average, the weight of the adult human brain is 1,400 g, ranging between 1,200 and 1,600 g. A smaller percentage of weight values above or below these (rarely over 2,000 g) is observed, with ample variation between individuals. In 1,130 adult and old age individuals of both genders, brain weight varied from 1,720 to 900 g (a variation of ~95%). This percent variation is related in part to gender (higher size and weight values in males, representing 9–12%) and body height^6,7^.

Starting at age 65, there is a 10/% reduction in average brain weight (and volume), with substantial individual variation. There is also a reduction in neuron size and dendritic arborization responsible for neuronal connectivity, although the number of neurons remains unchanged^6^. A reduction of 0.45 to 0.5% in brain volume per year has been reported in other studies, starting at the age of 60 years, as well as a reduction in thickness of the cerebral cortex, and partial loss of axon myelin sheath^8^.

A study conducted with 42 chimpanzees raised in captivity (adolescents, adult, and aged, males and females) showed that an average brain weight of 389,9 g is reached in adolescence (ages 7 to 15 years), practically the same as in young adults (ages 15–30 years), in which average brain weight is 390 g. Individual variations have also been reported (458 to 308 g, a variation of almost 50%), as well as a reduction in brain weight with age^9^. In humans, the average brain weight (around 1,400 g) represents 2% of the body weight for a 70-kg individual, whereas in chimpanzees, brain weight averages 390 g, corresponding to approximately 0.7% of the average body weight for an adult male individual of 54 kg (Table 1).

**Table 1:** Average brain weight, average number of cerebral cortex neurons, and energy consumption by the brain of chimpanzees, *Homo habilis*, *Homo erectus*, and *Homo sapiens.*

	Chimpanzee	*Homo habilis*	*Homo erectus*	*Homo sapiens*
Brain weight (g)*	390 (0.7%)	600-640+	900-1,041+	1,400 (2%)
Number of neurons (billions)	7.4	11	17	24
Energy consumption (%)§	9-13		16	19-27

Notes:

* Adult individual;

+Cranial capacity in cm^3^; ^§^% of energy consumption by the body at rest. The number of neurons in the human cerebral cortex (24 billion) estimated with basis on the rules of proportionality to the number of neurons in the various brain structures of primates is much higher than the average obtained by counting with the isotropic fractionator method (10.2 and 16.3 billion) and also when stereological methods are used (16.5 and 21 billion). See Table 2.

**Table 2:** Recent neuron counting studies of the human cerebral cortex.

Author	Year	Number of cases	Age (years)	Method	Number of neurons (billions)	Average (billions)
Haug H	1997	111	20-110	S	10-25	16.5
Pakkenberg B, Gundersen H	1997	94	18-93	S	14.7-32	21
Azevedo FA et al.	2009	4	50-71	IF		16.3
Castro-Fonseca E et al.	2023	43	25-87	IF	7.2-14.4	10.2*

Notes:

Abbreviations: S, Stereological; IF, Isotropic fractionator.

* The cerebral cortex contains 23.8 billion non-neuronal cells, most of which are glial cells;

See references.

To compare the modern human brain with the brains of our human ancestors, brain volume is estimated based on the cranial capacity of fossilized skulls, which is the volume of the inner space of the skull occupied by the brain. Comparison of the cranial capacity of *Homo sapiens* (1,350 cm^3^) with that of some of our ancestors shows progressive volume increase during evolution: *Australopithecus afarensis* (413 cm^3^), *Homo habilis* (600-640 cm^3^), and *Homo erectus* (900-1,041 cm^3^)^10^.

### Number of cerebral cortex neurons

Knowledge of the cellular constitution of the brain is very important for understanding its structure and function. Differences in nerve system organization in terms of the number of cells in a region or structure, cellular architectural arrangement, and proportion to which a region or structure is dedicated to a particular function can provide clues that help understand how the brain is organized and how it functions^11^.

The human brain has in average 86 billion neurons (NeuN-positive cells) and 84 billion non-neuronal cells (NeuN-negative cells), most of which are glial cells^12^. Of the total number of neurons, 16 billion (19%) are in the cerebral cortex. This proportion is similar to that observed in most mammal species (12 to 25%). Three studies conducted by different authors with a variable number of adult and aged individuals of both genders, using the (optical dissector) stereological and isotropic fractionator methods, showed that the cerebral cortex contains between 10–25, 14.7–32, and 7.2–14.4 billion neurons, respectively, the average number being 16.5, 21, and 10.2 billion, respectively. There was ample individual variation (100% or more) in all three studies, with no differences between males and females^6,13,14^, and no reduction in neuron numbers with age^6,14^. A neuronal loss of about 10% was reported by Pakkenberg and Gundersen^13^ (Table 2), yet no histopathological study was made by these authors to exclude the presence of neurodegenerative diseases that could explain neuronal loss.

The number of neurons in the cerebral cortex of humans is higher than that of chimpanzees (7.4 billion, according to a study conducted with a single female chimpanzee^15^). An estimation of the number of cortical brain neurons in human ancestors based on the rules of proportionality to the number of neurons in the various cerebral structures of primates shows that the cerebral cortex of *Australopithecus*, *Homo habilis*, and *Homo erectus* has respectively 9 billion, 11 billion, and 17 billion neurons, whereas the cerebral cortex of *Homo sapiens* has 24 billion neurons^16,17^. This neuronal number estimate for the human cerebral cortex is far larger than the average number obtained by counting with the isotropic fractionator method (10.2 and 16.3 billion) and by stereological methods (16.5 and 21 billion).

### Energy consumption by the brain

The brain consumes a high amount of energy, requiring continuous access to glucose and oxygen transported by the cerebral blood flow, which in humans represents 16% of cardiac output (the amount of blood pumped by the heart in 1 minute). Energy consumption by the brain varies according to age and gender, with larger amounts being consumed by young adults and women, and smaller amounts by men and the aged^18^. The extent to which the brain depends on constant blood supply is clearly demonstrated when a person suffers cardiac arrest, commonly secondary to myocardial infarction. Loss of consciousness occurs within approximately 15 seconds and, if cardiac reanimation is delayed, oxygen-glucose deprivation will cause neuronal death in several regions of the brain, particularly the cerebral cortex, four to seven minutes after cardiac arrest.

Compared to the great apes, humans consume more energy and have a faster basal metabolic rate, which may have favored evolution to a larger brain^19^. A though representing only 2% of body weight, the adult human brain consumes 19 to 24% of the amount of energy required by the human body at rest. This energy consumption rate is out of proportion to the relatively small mass of the brain^20^. The brain of adult great apes requires far less energy, with chimpanzees, for example, consuming 9 to 13% of its total. It is admitted that the high energy requirement of the human brain is attributable to its large number of neurons^17^.

A correlation is observed between, on the one hand, the areas of the carotid foramen in the skull and transverse foramen of the cervical vertebrae through which the main arteries that irrigate the brain pass –— the internal carotid arteries and the vertebral arteries, respectively — and, on the other hand, energy consumption by the brain, which seems to vary regardless of the number of neurons. To explain these findings, it has been proposed that a larger blood flow to the brain was required in humans, not because the brain became more expensive in terms of energy, but rather because, as our ancestors evolved, brain size increased in relation to body size^21^.

### Anatomical and functional asymmetry

Lateralization of the brain, with some brain functions tending to be served by only one of the brain hemispheres, is present in several animal species and probably reflects an ancient evolutionary division between the two hemispheres of functions that are essential for survival, involving sensory and motor aspects^22,23^. In modern humans, lateralization provided a solid neural basis for the development of higher motor and cognitive functions, such as using the hands as a tool, resulting in handedness (right-handed and left-handed individuals, depending on which hand is preferably used), as well as lateralized brain control for speech perception and production. These, in turn, led to the development of technology and more efficient forms of communication and social interaction, respectively.

The left hemisphere of the brain has dominance for language, particularly in right-handed individuals. In 90-95% of humans, it is also dominant for using the right hand for gestural communications and tool manipulation. Most left-handed individuals (78%) also show dominance of the left hemisphere for language. The right hemisphere is dominant for music production and visual spatial integration, which is the ability to represent objects in three dimensions and, thus, estimate the distance between them^24^. Even though symbolic language is a cognitive and behavioral innovation unique to the human species, lateralization of the cortical areas associated with language in humans is also found in great apes, which suggests that language lateralization was already present in our last common ancestor.

The main anatomical asymmetries in the human brain are brain torque, *planum temporale*, Broca area, and arcuate fasciculus (which are related to language evolution and speech production and perception), and the fusiform gyrus, involved in facial recognition.

### Brain torque

This consists of counterclockwise torsion of the cerebral hemispheres, resulting in brain shape asymmetry, characterized by several aspects^25,26^ such as:

- petalias;
- downward displacement of the left occipital pole relative to the right occipital pole;
- right-slanting of the medial surface of the occipital lobe;
- asymmetry of the cerebral hemispheres in length, height, and width.

Right-frontal and left-occipital petalias, which are present in 60% of the human population, consist of forward displacement of the right-frontal pole and backward displacement of the left-occipital pole, commonly observed in anatomical and neuroimaging studies. Petalias of a similar or inverse pattern are identifiable in human ancestors^10^.

*In vivo* analysis using three-dimensional structural magnetic resonance imaging of the brains of 91 humans and 78 chimpanzees showed that brain torque is specific of humans and absent in chimpanzees. In addition, the left hemisphere of the human brain is lower and longer than the right hemisphere (lengths of 173.8 and 172.9 mm, respectively), whereas the cerebral hemispheres of chimpanzees have equal dimensions. It is admitted that these cerebral asymmetries in humans may potentially correlate with the evolution of language^27^.

### Planum temporale

This cortical area is in the lateral sulcus, posteriorly to the primary auditory cortex on the superior temporal gyrus and is associated with language comprehension. In 65 to 70% of individuals, the left *planum temporale* is larger than its counterpart on the right, as demonstrated by Geschwind and Levitsky in a 1968 study of a sample of 100 brains. This asymmetry is shared by humans and all great apes^28,29^.

A study of the cytoarchitecture of the left *planum temporale* showed that its minicolumn arrangement, with a larger spacing between minicolumns, as compared to its counterpart on the right, in which the minicolumns are less spaced, is responsible for the asymmetrical cortical surface of the human *planum temporale*. This asymmetry is not present in chimpanzees (see review^30^). A larger spacing between minicolumns equals a larger amount of neuropil, which, in turn, represents a higher number of dendrites, axon terminals, and synapses, allowing for more refined processing by the minicolumns^31-33^.

A recent study of 98 individuals using structural magnetic resonance and diffusion tensor imaging during auditory perception of speech as reflected by electroencephalography showed that the volume of the intra-neurite fraction, which indicates the density and spatial dispersion of dendrites and axons, was larger in the left *planum temporale* as compared to its right-size counterpart (2,081 mm^3^ x 1,645 mm^3^, respectively)^34^. This means that the higher density and spatial dispersion of dendrites and axons in the left-right *planum temporale* are associated with faster neural processing of auditory speech perception.

### Broca area

Located in the left inferior frontal gyrus, Broca area contains the speech motor programming (articulatory code) that is relayed to the laryngeal motor cortex located in the primary motor cortex behind Broca area, to produce phonation in the larynx. In bonobos and chimpanzees, too, vocalization is lateralized in the left cerebral hemisphere. The volume and number of neurons in Broca areas 44 and 45 of both cerebral hemispheres were analyzed in a small sample of 10 adult humans (5 males and 5 females). In all 10 cases, the volume of area 44 was larger in the left hemisphere than in the right hemisphere, although this asymmetry was significant only in males. In 6 of these individuals (including all females), the volume of area 45 was significantly larger in the left than in the right hemisphere^35^.

In males, area 44 of the left hemisphere had a larger number of neurons than its counterpart on the right. Although the total number of neurons in area 45 on the left side was larger than that on the right side in all 5 females, the difference was not significant. In males, there was no difference between the cerebral hemispheres in the total number of neurons in area 45. New studies with a larger sample of individuals are necessary to corroborate these findings.

The cytoarchitecture of Broca area in humans and in its homologue in chimpanzees and bonobos exhibits large pyramidal neurons in layers III and V in all species studied. However, differences between the species are observed in the volume occupied by the cell body of neurons and neuropil in the six cortical layers. Proportional neuropil volume relative to cell body volume, which reflects the number of dendrites, axon terminals, and synapses required for information processing between neurons, is larger in humans as compared to great apes^36-40^.

### Arcuate fasciculus

This important curve-shaped (hence "arcuate") fasciculus connects areas of the inferior frontal and lateral temporal areas, including Broca and Wernicke areas responsible for speech and comprehension of spoken and written language. A direct connection between Broca and Wernicke areas by the arcuate fasciculus is present in the left cerebral hemisphere of all humans. In the right cerebral hemisphere, however, this connection is robust in 17.5% individuals, less robust in 20% of individuals, and absent in the remaining 62.5%^41,42^. It is worth noting that, in both cerebral hemispheres, an indirect connection between Broca and Wernicke areas is provided by the arcuate fasciculus via an intermediary connection on the angular gyrus of the inferior parietal lobule.

Projection of the three subdivisions — fronto-parietal, temporal-parietal, and fronto-temporal — of the arcuate fasciculus is well developed in humans, but quite reduced in chimpanzees^43-46^.

### Fusiform gyrus

The human right fusiform gyrus (in the lower portion of the temporal and occipital lobes) is dominant in face recognition^47^. While integral, holistic face processing is done in the dominant right fusiform gyrus and shared by chimpanzees (although not lateralized as in humans), processing of individual facial features takes place in the left fusiform gyrus^48^.

An interesting fact about the left fusiform gyrus is that its neurons are larger, its minicolumns are wider, and its neuropil is more voluminous, as compared to those of the right fusiform gyrus dominant for face perception. It seems, therefore, that the thinner minicolumnar arrangement and less neuropil in the right fusiform gyrus are what make it dominant for configurational face processing. This asymmetric cortical architectural pattern of the fusiform gyrus is not observed in chimpanzees.

### Cerebral cortex expansion

Expansion of the cortical area of the brain, especially the neocortex, occurred in all mammals, although it was particularly more intense and faster in primates. This expansion was both in absolute terms and relative to the rest of the brain, causing the human brain to acquire more folds and grooves, with the gyri becoming more tortuous^14,49,50^.

In addition to the primary sensory and motor areas shared with other mammals, the cerebral cortex of primates acquired new cortical association areas (distinct in terms of anatomy, cytoarchitecture, and function) that were involved in higher order processing, allowing for the integration of two or more senses (sight, hearing, body position in space), known as multimodal integration, and the generation of complex movements^51^.

With regard to cortical cytoarchitecture, for example, the neurons in association areas exhibit a larger number of synapses and a more complex dendritic arborization. The superior and inferior parietal lobules expanded greatly to include sensory-motor modules that process and integrate information into motor commands, such as adjusting and fixing the eyes when we see something interesting, reaching, grabbing, and self-defense, allowing humans to acquire new skills, such as manipulating tools and using gestures for communication. A large cortical expansion occurred in the temporal-parietal junction and posterior superior temporal sulcus, which contain areas involved in emotional face expression recognition, attention orientation, theory of mind (the ability to infer the mental state of others), and memory. This allowed rearrangement of the cortical areas located in these regions and the appearance of new functional cortical areas, contributing to an increase in human social skills^52^.

The primary motor cortex, which is responsible for muscle action, became more specialized in the use of arms, hands, and fingers, as well as in voluntary actions while walking, providing more advantages to primates, especially humans. These functions were further heightened by addition of the frontal premotor and motor cortical areas of the anterior cingulate cortex, which are involved in movement planning. The prefrontal cortex in humans expanded greatly in relation both to that of great apes and to the other regions of the brain, contributing to cognitive emotional control and social behavior and decision-making modulation^53^.

The large expansion of the cerebral cortex in humans in relation to non-humans and other mammal groups, unaccompanied by a significant increase in cortical thickness, is attributable to an increase in the number of minicolumns^54^. In humans, there has also been an increase — in absolute terms and relative to the other cortical layers — in the thickness of layers II and III, in dendritic arborization size and complexity, as well as in the size of the pyramidal neuron spines in these layers, with expansion of the cortico-cortical connections and improvement of the information processing and integration capacity of these connections^55^.

In short, expansion of the cerebral cortex was caused by an increase in the number of neurons and synapses, particularly in humans, in whom the neocortex occupies approximately 40% of the total brain volume, a much larger proportion than that expected for the body weight of primates. Cerebral cortical expansion in humans has been to a large extent responsible for many of the behavioral specializations that are unique to our species, such as fine hand motor control, cognitive and social flexibility, and language production and comprehension, allowing humans to process and represent information regardless of sensory stimulation, *i.e*., our brain is capable of generating events that are not taking place in the environment — the essence of abstract thinking.

### Prefrontal cortex

As mentioned earlier, the most recently evolved and largest portion of the frontal lobe occupies the entire front part of the frontal lobe — hence the name prefrontal cortex — and is one of the most important regions of the human brain. The prefrontal cortex is associated with cognitive and behavioral functions, emotional-cognitive integration, working memory, executive functions, decision-making, long-term planning, delayed gratification, and impulsivity control^56-60^.

Based on spatial location along the various geometric planes — superior, inferior, anterior or ventral (front), posterior or dorsal (back), lateral, medial — the prefrontal cortex is divided into various areas with distinct functions: ventromedial prefrontal, ventrolateral, dorsomedial, dorsolateral, frontopolar (frontal pole), orbitofrontal (above the orbits), and anterior cingulated cortex (on the medial face of the cerebral hemispheres, above the corpus callosum)^2,61^.

### Volume of the cortical grey matter and subcortical white matter of the prefrontal cortex of non-human primates and humans

A study of 60 humans, 29 chimpanzees, and 19 Old World monkeys showed that the volume of the cortical grey matter of the human prefrontal cortex (which contains neuron cell bodies, glial cells, and neuropil) and subcortical white matter was 1.9 times larger as compared to monkeys and 1.2 times larger as compared to chimpanzees. The subcortical white matter (containing nerve fibers entering and leaving the prefrontal cortex) was 2.4 times larger in humans as compared to monkeys and 1.7 times larger as compared to chimpanzees^53^. These results suggest that the larger volume of the human prefrontal cortex is due not only to its greater number of neurons (see below), but also to its larger number of cortical synaptic connections, (probably) higher number of axons per unit of area, and/or a larger axon diameter.

### Number of neurons in the human prefrontal cortex

The frontal lobe contains 34% of the cerebral cortex neurons, of which 85% are in the prefrontal cortex and 15% in the primary motor cortex (on the precentral gyrus)^14^. These data indicate that, as the frontal lobe is the largest region of the brain, the prefrontal cortex is the brain area with the largest number of cerebral cortex neurons. It should be noted that the premotor and supplementary motor cortices were considered by the authors of this study as being part of the prefrontal cortex.

### Cytoarchitecture of the prefrontal cortex in non-human primates and humans

A comparison of the cerebral cortical cytoarchitecture of humans *versus* great apes shows that Broca area and the anterior insular cortex of humans are 6 times larger than their homologues in chimpanzees, whereas the primary motor cortex, primary somatosensory cortex, and primary visual cortex have similar sizes in both species^62^. These findings are in agreement with those from other studies reporting thicker minicolumns and a larger amount of neuropil in layer III of the human frontopolar cortex (indicating more refined neural processing) as compared to chimpanzees^63^.

Similarly, the number of spines in the pyramidal neurons of the human prefrontal cortex, which points to the existence of synapses, is also larger as compared to non-human primates, thus further supporting the point of view that there was expansion of the prefrontal cortex in humans^64^.

### Connectome

The different types of neurons and their synaptic connections at the micro- and macroscopic levels make up the neural networks known as connectome. At the microscopic level, connections between the neurons are made by synapses that convey information by means of neurotransmitters. These synapses, identifiable under an electronic microscope, are collectively known as the synaptome^65,66^. Studying the microscopic connections of our nerve system is important, as the individual neural identity of humans is thought to be defined by our connectome. In other words, humans are different, feel and think differently from other beings because the neural synaptic connections are distinct and unique to each of us, even though the human brain is admitted having hundreds of trillions of synapses.

Three-dimensional macroscopic visualization of the connectome is possible using special magnetic resonance techniques, such as diffusion tensor imaging combined with tractography, allowing identification of the dominant orientation of the nerve fibers that form the white matter tracts. In 2009, the U.S. National Institutes of Health launched the *Human Connectome Project* to initially map the brains of 1,200 adults using the latest imaging technology. The data from this project are available on-line for laboratory analysis by neuroscientists all over the world.

Over the past two decades, there has been a boom of *in vivo* studies of individuals, including humans and non-human primates, using structural and functional magnetic resonance and tensor diffusion imaging to map distinct regions of the brain and their connectivity patterns. Comparative analyses of the human connectome *versus* that of other species showed that the organization and spatial distribution of cortico-cortical and cortico-subcortical fibers are similar in monkeys, chimpanzees, and humans. This similarity is attributed to selective pressures common to these primates, leading to a reduction in connectivity costs and, at the same time, to more complex adaptive functions. Quatitative differences, as well as differences in connectivity patterns, are observed among the groups of primates.

Several neural networks show similarity to those of humans, such as the corticospinal tract (which extends from the cerebral cortex to the spinal cord motor neurons responsible for the execution of muscle actions), connections of the limbic system involved in social behavior and emotion modulation (cingulum and fornix bundle), and fasciculi that interconnect areas of the different brain lobes, allowing processing and integration of information from association and primary sensory and motor areas and forming an ample neural network related to attention control^45^.

Among these fasciculi, the following stand out:

- superior longitudinal fasciculus, which connects the lateral frontal cortex to the lateral parietal cortex;
- inferior longitudinal fasciculus, which connects the temporal to the occipital lobe (the longitudinal name of these two fasciculi derives from their direction relative to the main axis of these lobes)^67^;
- inferior fronto-occipital fasciculus, which connects the occipital lobe and, to a lesser degree, the parietal and temporal lobes to the frontal lobe^68^.

It is admitted, for example, that the inferior fronto-occipital fasciculus is related to visual information processing and integration for basic communication acts between individuals of the same species, such as producing distinct screams to alert when threatened by different predators.

Significant differences among primate species are found, however, in connectivity of the multimodal association areas, which is more elaborate in humans as compared to chimpanzees^43^. Examples are connections of the lateral parietal cortex, precuneus (superior, medial, and posterior portions of the parietal lobe), temporal cortex, inferior frontal cortex, and medial prefrontal cortex, including important language processing tracts such as the arcuate fasciculus, which, as mentioned before, connects Broca and Wernicke areas responsible for speech production and spoken and written language comprehension^42,44^.

During evolution, there has been a significant increase in connectivity of the posterior middle temporal gyrus and anterior portion of the left temporal lobe in humans as compared to chimpanzees. The posterior middle temporal gyrus is involved in meaning attribution to words (semantics) and syntax processing (organizing words into phrases and sentences), whereas the anterior portion of the temporal lobe is associated with semantic representations of language. Connectivity of the posterior middle temporal gyrus was expanded in all three subdivisions of the arcuate fasciculus, particularly the parieto-temporal subdivision, as well as in the middle longitudinal fasciculus. On the other hand, connectivity of the anterior portion of the temporal lobe of humans also expanded in the inferior longitudinal fasciculus, middle longitudinal fasciculus, and uncinate fasciculus, the latter of which connects the anterior portion of the temporal lobe to the orbitofrontal cortex^45,46^.

Another important difference between humans and non-human primates is in the motor control of speech and vocalization by the laryngeal motor cortex. In humans, the laryngeal motor cortex is in two areas of the primary motor cortex (precentral gyrus): the face representation area (dorsal laryngeal motor cortex) and the area underlying it (ventral laryngeal motor cortex). The neurons of the laryngeal motor cortex make direct connections via the corticobulbar tract with the motor neurons in the nucleus ambiguus (located in the brain stem), facilitating control of the laryngeal muscles and complex movement production in speech and singing^69,70^. Compared to non-human primates, the dorsal laryngeal motor cortex shows connectivity and synchronicity with motor control of the jaw, (especially for lowering of the jaw) during phonation^71^. There is also greater connectivity with the adjacent temporoparietal cortex (inferior parietal lobule and superior temporal gyrus) responsible for more refined sensory and motor processing and execution, which may have contributed to better sensory and motor integration, resulting in more elaborate motor control of learned vocalizations such as speech and singing.

In non-human primates, the laryngeal motor cortex is in the premotor cortex and its neurons make only indirect connections with the nucleus ambiguus, restricting the production of vocalization patterns learned voluntarily.

Also, there is preference for the human left cerebral hemisphere to interconnect exclusively with itself, particularly with the cortical areas involved in language and fine motor coordination, the latter being related to using the hands to thread a needle, play a musical instrument or dissect an anatomical structure using a scalpel. In contrast, the cortical areas of the human right cerebral hemisphere associate with visual-spatial processing and attention interact with both cerebral hemispheres^72^.

Differences are also observed in the organization of the superior longitudinal fasciculus, which, as mentioned before, connects the lateral frontal cortex with the lateral parietal cortex, the latter being considered by some anatomists to be distinct from the arcuate fasciculus. In humans, as compared to chimpanzees, there is greater connectivity of the superior longitudinal fasciculus with the lateral frontal cortex (dorsolateral prefrontal cortex and inferior frontal gyrus), more to the right in the latter, thus evidencing lateralization^73^. These differences may have implications for the evolution of some functions related to the frontal and parietal lobes, such as attention for comprehending and reproducing the details of an observed action, social learning, and use of tools.

A reduced proportion of connections (5.9%) seem to be specific to the human connectome, forming the intra-hemispheric pathways (connecting different areas of the same cerebral hemisphere) related to multimodal, unimodal, and primary (sensory and motor) association areas, with connections among these areas also being present^43^. Of the cortico-cortical intra-hemispheric connections in the left cerebral hemisphere, 82.6% (19 out of 23) are specific to humans and 50% (3 out of 6) are specific to chimpanzees. As compared to the connectome of chimpanzees, the human connectome exhibits relatively high levels of integration among functionally segregated brain systems, which allows for greater precision in making a correct choice^74^.

Based on data from the *Human Connectome Project* and functional magnetic resonance studies, new cortical and subcortical functional neural networks have been described as associated to:

- spatial navigation and identification and representation of events in a narrative;
- semantic categories of a higher order, such as in processing and integrating meaning to a spoken, heard or seen object (or another category); and
- reward system^75^.

Individual differences are identified in the functional connectome of humans at rest, which shows that some neural networks are unique and stable, functioning as a neural fingerprint^76^.

Provided below is a summary of the quantitative anatomical changes in the human brain that have made it unique and allowed for the appearance of refined (*) and new (+) cognitive and behavioral aspects, as compared to chimpanzees and bonobos — the great apes that are our closest relatives from an evolutionary point of view — and our human ancestors (Chart 1)^2,77^.

**Chart 1:** Quantitative evolutionary changes in the anatomy of the human brain that resulted in its uniqueness.

Absolute increase in brain size and in the number of neurons in the cerebral cortex; Anatomical and functional asymmetry; Expansion of the cerebral cortex in the association areas; More diversified cytoarchitecture of the cerebral cortex, with hemispheric asymmetry; Appearance of refined* and new+ cognitive and behavioral aspects: Reduction in reactive aggressiveness* Communication and cooperation for hunting, defense, and tool use* 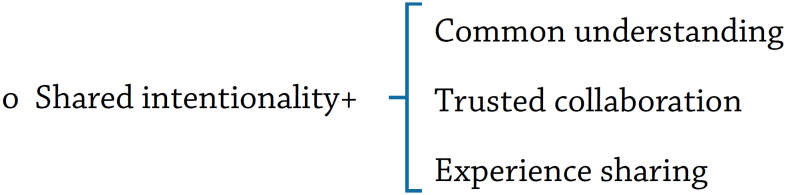 Symbolic articulated language+ Formation of larger social groups and creation of artifacts, trade, art, science, philosophy and religion, allowing for cumulative cultural evolution+ Examples of shared intentionality (collective work or activity performed jointly by a group of individuals): sports teams, musicians of an orchestra, production of an opera, production of a film with actors, a military or samba school parade.
